# Fast, linked, and open – the future of taxonomic publishing for plants: launching the journal PhytoKeys

**DOI:** 10.3897/phytokeys.1.642

**Published:** 2010-11-01

**Authors:** Lyubomir Penev, W. John Kress, Sandra Knapp, De-Zhu Li, Susanne Renner

**Affiliations:** 1 Bulgarian Academy of Sciences & Pensoft Publishers, Sofia, Bulgaria; 2 Smithsonian Institution, Washington DC, USA; 3 Natural History Museum London, UK; 4 Kunming Institute of Botany, Chinese Academy of Sciences, Heilongtan, Kunming, Yunnan 650204 China; 5 University of Munich (LMU), Germany

**Keywords:** E-publications, open access, semantic tagging, semantic enhancements, *plant systematics*

## Abstract

The paper describes the focus, scope and the rationale of PhytoKeys, a newly established, peer-reviewed, open-access journal in plant systematics. PhytoKeys is launched to respond to four main challenges of our time: (1) Appearance of electronic publications as amendments or even alternatives to paper publications; (2) Open Access (OA) as a new publishing model; (3) Linkage of electronic registers, indices and aggregators that summarize information on biological species through taxonomic names or their persistent identifiers (Globally Unique Identifiers or GUIDs; currently Life Science Identifiers or LSIDs); (4) Web 2.0 technologies that permit the semantic markup of, and semantic enhancements to, published biological texts. The journal will pursue cutting-edge technologies in publication and dissemination of biodiversity information while strictly following the requirements of the current International Code of Botanical Nomenclature (ICBN).

## Introduction

Exciting and novel advances in the publishing and dissemination of taxonomic information are changing the field. The appearance of electronic media as conduits of scientific communication and the adaptation of the Internet as a medium of transmission and dissemination means that taxonomists, publishers, and indexing and aggregation services have the chance to use these new tools to accelerate biodiversity research and understanding. Accompanying these changes has been the development of methods to increase the speed and efficiency of sampling of biological materials and discovery of new taxa, thanks to the development of new methods, especially the application of DNA sequencing to taxonomic work.

The challenges are indeed daunting in scale. From the viewpoint of biodiversity publishing, these challenges could be summarized in four main groups: (1) Appearance of electronic publications as amendments or even alternatives to paper publications; (2) Open Access (OA) as a new publishing model; (3) Linkage of electronic registers, indices and aggregators that summarize information on biological species through taxonomic names or their persistent identifiers (GUIDs, currently LSIDs, Life Science Identifiers); and (4) Web 2.0 technologies that permit the semantic markup of, and semantic enhancements to, published biological texts.

In response to these publication challenges, we are here establishing a new journal in plant systematics, called PhytoKeys. PhytoKeys (http://www.phytokeys.com) builds on existing experience and innovations accumulated during the successful launch of its partner journal ZooKeys (http://www.zookeys.org). PhytoKeys aims to set new standards in taxonomic publishing and especially dissemination, in full compliance with the current International Code of Botanical Nomenclature (ICBN).

## E-publish or perish? Print and electronic publications of nomenclatural acts and biological Codes

The future of biodiversity publishing in the digital era has provoked lively discussions in the last few years, most of them focusing on the permissibility of electronic publication of nomenclatural activities, such as new species descriptions or lectotypifications. Were electronic publication to be allowed, the biological Codes would need revision. Both, the International Code of Botanical Nomenclature, ICBN (Article 29, Recommendation 30A), and the International Code of Zoological Nomenclature, ICZN (Articles 8 and 9), currently do not allow strictly e-only publications of nomenclatural acts (for the ICZN, see details in Knapp and Wright 2010).

Effective publication under the ICBN is currently defined “*only by distribution of printed matter (through sale, exchange, or gift) to the general public or at least to botanical institutions with libraries accessible to botanists generally*“ (Article 29). At the International Botanical Congress in Vienna in 2005, Recommendation 29A laid out a preliminary set of ideas about the relationships between print and electronic versions of an article:

“Publication of nomenclatural novelties in periodicals ....... that distribute an electronic version as well as a printed version, should only be in those with the following features:

1. The printed and electronic versions are identical in content and pagination;
2. The electronic version is in a platform-independent and printable format;
3. The electronic version is publicly available via the World Wide Web or its successors;
4. The presence of nomenclatural novelties is prominently indicated in the work ….”

(Recommendation 29A, ICBN)

In our view, best practice for any journal currently publishing nomenclatural information electronically should consider the following criteria to ensure effective publication:

1. Maintenance of a printed version registered under print ISSN (P-ISSN), different from the ISSN of the electronic version (E-ISSN);
2. Production of the print version simultaneously with the electronic version;
3. The printed version to be identical (including resolution and color) to the electronic (normally PDF) version;
4. Maintenance of a stock of the printed version that may be requested and delivered on purchase, exchange or gift;
5. Publication of the electronic version on the World Wide Web.

Descriptions of new taxa are already being published in e-only journals and authors and publishers have carefully followed the current requirements of the Codes. New plant taxa published recently in an entirely electronic journal, PLoS One (Knapp 2010) accomplished effective publication of the names therein by the authors themselves taking care to print the articles and send them to various libraries to provide paper archiving. It is obvious that such a policy intended to satisfy the Codes is not sustainable on the long term and makes changes and amendments to both Codes like those that have been suggested recently more topical and urgent (Knapp et al. 2007, Knapp and Wright 2010, Knapp et al. 2010, Wheeler and Krell 2007). Amendments to the Codes are currently under active discussion in both the zoological and botanical/mycological communities (e.g., Availability & electronic publication 2010, Chapman et al. in press).

The policy of PhytoKeys regarding electronic publication is very clearly defined. We shall strictly follow the requirements of the current International Code of Botanical Nomenclature (Vienna Code), adopted by the Seventeenth International Botanical Congress Vienna, Austria, July 2005 (http://ibot.sav.sk/icbn/main.htm). The journal will be published simultaneously in online and print formats. The high-resolution, full-color print version is identical to the online PDF version. In addition, the entire content of the journal is published open access, free to anyone to download, archive, print and distribute.

## Publish for free or read for free? Open access in biodiversity publishing

PhytoKeys is established as an entirely open access journals and adheres strictly to the principles of free exchange of knowledge, which means a direct, barrier-free, online dissemination of scientific results at no charge to the reader (see the Berlin (2003) and Bethesda (2003) Declarations on Open Access). Under the open access model and according to the Creative Commons Attribution License (CC-BY) used by PhytoKeys, authors retain the rights for their articles, which may however be copied, downloaded, and used for text- and data-mining purposes, provided that such uses are fully attributed to the author(s) and source of publication.

By publishing open access, authors benefit from a higher visibility and increased citation rate of their papers; analyses of articles published between 2001 and mid-2009 (Wagner 2010) found 39 cases of an open access citation advantage (OACA) and 7 showing either no OACA effect or ascribing OACA to factors unrelated to OA publication. Open access articles are downloaded more than articles for which subscriptions fees must be paid; Wagner (2010) concluded that “*studies typically show a 25–250 % OACA or more. The higher end of that range might prove illusionary. However, even if the true OACA turns out to be only 10–15%, this would still be a major incentive for scholars to choose an open access publishing option.*”

Another important advantage to open access publishing is that it permits immediate, often automated, distribution of the published contents to bibliographic databases, e-archives, indexers and aggregators [e.g., Encyclopedia of Life (EOL), Global Bidoidversity Information facility (GBIF), PubMedCentral, Wikispecies, Wikipedia, Wikimedia, Plazi, and many others). Furthermore, the published text can be “atomized” and disseminated in fragments associated with bibliographic metadata. For instance, taxon descriptions and associated discussions can be automatically supplied through the Species Profile Model (SPM) to EOL (http://wiki.tdwg.org/twiki/bin/view/SPM/PlaziEOLProject) and locality data supplied to GBIF through the Integrated Publishers Toolkit (IPT) (Chavan and Ingwersen 2009, Penev et al. 2009b).

As a business model, open access (“author pays but everyone can read at no charge”) is often opposed to conventional publishing model (“publisher pays but everyone has to pay to read”) (Suber 2003, 2007). Publication fees in open access journals ensure a barrier-free distribution of the contents and include costs involved in processing, formatting, publishing, indexing, and archiving of the published materials. It is expected that authors cover the open access fee from institutional funds or from grants from funding agencies. The current policy of funding bodies in many developed countries is to provide grant money for open access publishing in their budgets. For instance, such major funding bodies as the European Union’s Framework Program Seven (FP7), National Institutes of Health (NIH) of the USA, and Welcome Trust in UK already demand open access of the published scientific results they fund (see for instance the Open Access Pilot in FP7 (2009) or SPARC Europe News (2007).

Unfortunately, some authors of worthy manuscripts may be constrained in their ability to pay open access fees, e.g., students, scientists from developing countries, or retired scientists. In PhytoKeys, such authors will have the option of discounted fees or a complete waiver. Discounts or waivers will also be offered to scientists who actively participate in the review and editorial process. We hope with these incentives to give all botanists the opportunity to experience the pleasure and benefits derived from open access publishing.

Although open access is not yet entirely accepted in taxonomy publishing due to several constraints (Agosti and Johnson 2006), its value is clear and the proportion of electronically published taxonomic work is rapidly increasing. In addition, there is a growing demand that at least descriptions of taxa should be placed firmly in the public domain (Agosti and Egloff 2009). The revolutionary changes occurring in the transition to Web 2.0, whereby all publications can be linked to form one virtual entity (rather than thousands of individual entities – see Agosti et al. 2007) will greatly increase the importance of open access publications. The mission of PhytoKeys is to further progress in this direction.

## Publish “indivisible” entities or “atomize” content? Semantic markup of and semantic enhancements to taxonomy publications

Scientific publishing for over 500 years has developed around two basic models: (i) the large scientific monograph and (ii) the scientific article within a periodical (journal). Both models have traditionally been seen as producing “indivisible” entities of published information, identified, described and cited through their bibliographic descriptions (today called “metadata”). Division, separation and analysis of the body texts of scientific publications have been possible only through reading on paper by humans.

The Internet and especially Web 2.0 technologies, also known as the Semantic Web (http://en.wikipedia.org/wiki/SemanticWeb), have stimulated the development of radically new models of publication, dissemination, reading and analysis of scientific content. These innovations have occurred with unprecedented speed and scale, and have already visibly impacted taxonomic publishing (for a review, see Penev et al. 2010a). We are on the verge of being able to have scientific texts read, harvested, and sorted out in databases entirely by computers. A key requirement for the success of these models is standardized methods and protocols for text processing and their implementation in routine editorial practices. Semantic mark up, or tagging, is a method that assigns markers, or tags, to text strings such as taxonomic names, gene sequences, localities, designations of nomenclatural novelties and so on. Tags “translate” the meaning of the respective strings into machine-readable languages like XML (eXtensible Markup Language). Semantic tagging allows not only computerized methods of archiving and data mining from articles but it also provides the basis for so-called “semantic enhancements”, defined as “anything that enhances the meaning of a published journal article, facilitates its automated discovery, permits its linking to semantically related articles, provides access to data within the article in actionable form, or facilitates integration of data between articles” (Shotton et al. 2009). Semantic Web technologies represent a vast and dynamic area of development, and we do not aim to discuss them in detail here. Recently, the concept of semantic tagging and its potential for semantic enhancements to taxonomic papers have been reviewed and illustrated by working examples in a special issue of ZooKeys (Penev et al. 2010b). PhytoKeys will build on and further develop technologies described and implemented in ZooKeys.

PhytoKeys uses the Pensoft Mark Up Tool (PMT) as the basic software tool for XML mark up implemented within the editorial process. PMT is based on the TaxPub XML schema, an extension to the Document Type Definitions (DTD) of the US National Library of Medicine Journal Archiving and Interchange Tag Suite (NLM; http://sourceforge.net/projects/taxpub). PMT provides highly automated, fine granularity mark up, for example, denoting each separate taxon treatment within a paper, or tagging of all taxon names, literature references, gene sequences, etc. The final XML outputof the paper is validated against the National Library of Medicine (NLM) document type definition (DTD) and could be archived in PubMedCentral upon approval of the latter. The taxon treatments (including descriptions of new taxa) are exported through XML to Encyclopedia of Life (EOL), Plazi and other interested aggregators of information. The PDF file of a paper will be identical to the printed version and will be stored for browsing or searching through the Biodiversity Heritage Library (BHL). Papers will also be published as semantically enhanced HTMLs, allowing interactive reading and use by methods such as: (i) visualisation of main tag elements within the text (e.g., taxon names, taxon treatments, localities, etc.); (ii) internal cross-linking between paper sections, citations, references, tables, and figures; (iii) mapping of localities listed in the whole paper or within separate taxon treatments; (v) autotagging of taxon names, with dynamic links to a wider array of large international biodiversity databases through the Pensoft Taxon Profile (PTP) (see next section for details), (vi) autotagging of GenBank and Barcode of Life Database (BOLD) accession numbers and linking to NCBI and BOLD, respectively; (vii) linking of taxon names to relevant references in PubMed, Google Scholar, Biodiversity Heritage Library, and other databases.

A substantial feature of the semantic Web is open data publishing, where not only analysed results, but original datasets can be published as citeable items. The incentives for authors and institutions to publish in PhytoKeys can be summarised as follows (Costello 2009, Smith 2009, Chavan and Ingwersen 2009, Penev et al. 2009a, Kühn et al. 2010):

1. Data produced and collected using public funds can be published, cited, used and re-used in the future, either as separate datasets or collated with other data;
2. Data can be indexed and made discoverable, browsable and searchable through biodiversity infrastructures (GBIF and others):
3. Data can be integrated with other dataset across space, time and taxonomic groups, bringing in this way recognition and new opportunities for collaboration to the authors;
4. Collection managers can trace usage and citations of digitized data from their collections;
5. By publishing data, authors and institutions are credited for their work to create and maintain datasets through:
  - Registering of priority and authorship in a conventional journal publication;
  - Indexing, discovery and citation in the same way as a standard research paper, to benefit authors in recognition and career building;
6. Datasets, metadata and respective data papers are inter-linked to expedite and mutually extend the dissemination, to the benefit of the authors and society.

PhytoKeys will support various methods for data publication. For instance, occurrence data sets can be published as downloadable files under a separate DOI number linked to the respective paper such as a taxonomic revision or floristic catalogue (for examples see Miller et al. 2009, Rasmussen and Asenjo 2009, Penev et al. 2009a). Identification keys can be published in several formats, from plain text formats for dichotomous keys, to HTML versions of the same key cross-linked to figures, references and individual couplets or DELTA, Lucid or MX interactive keys, published as downloadable primary files. The last option will allow future researchers to download and then modify keys by adding or removing taxa or diagnostic characters to adapt keys for local users (Sharkey et al. 2009, Penev et al. 2009b, Stoev et al. 2010).

A special feature of PhytoKeys will be the opportunity to publish data papers for datasets already uploaded and indexed by the authors to GBIF. Such data papers can be automatically extracted from the GBIF metadata catalogue, generated in a form of XML-tagged manuscripts through the GBIF’s Integrated Publishers Toolkit (IPT) and then submitted to PhytoKeys for regular review and editorial processing (http://data.gbif.org, see also Chavan and Ingversen 2009, http://www.cbd.int/gti/doc/gbif-IPT-en.pdf).

A challenging new approach that will be implemented in PhytoKeys is to streamline taxonomic publishing by handling manuscripts generated from authors’ databases or community websites, such as Scratchpads and LifeDesks. This process was recently prototyped and implemented in our sister journal ZooKeys (Blagoderov et al. 2010).

Semantic mark up and enhancements are expected to greatly extend and accelerate the way in which taxonomic information is published, disseminated and used. The mission of PhytoKeys is to launch a venue for botanists to use and enjoy these exciting opportunities in the rapidly changing world of publishing.

## Link yourself or perish: Electronic registers, indexers ad aggregators, or how to get linked to all?

**Figure 1.**
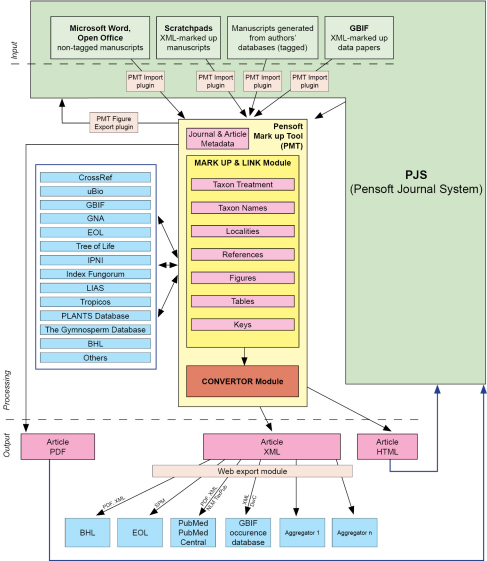
Editorial process in PhytoKeys based on XML mark up workflow and extensive internal and extrenal cross-linking to taxon databases, leading biodiversity platforms, indexers and aggregators.

The Semantic Web could also be called a “linked Web” because most semantic enhancements are provided through various kinds of links to external resources. Standard hyperlinks to resources such as DOI numbers for publications or GenBank accession numbers are already usual components of advanced journal publishing. There is, however, still much to be done in this direction, and the vision of PhytoKeys is to develop and implement new ways of cross-linking with specialized biodiversity resources. The external linking will be provided in-house, within the editorial process, so that the authors are not bothered with these sometimes quite cumbersome processes (Fig. 1).

The results of these linkages will be visualized in the HTML versions of the published papers through various cross-links within the text and more particularly through the Pensoft Taxon Profile (PTP) (http://ptp.pensoft.eu), a web-based harvester that automatically links any taxon name mentioned within a text to external sources and creates a dynamic web page for that taxon. PTP saves readers a great amount of time and effort by gathering for them the relevant information on a taxon from leading biodiversity sources in real time (Table 1). The PTP does not distinguish between names used in botany, mycology and zoology, so information on an animal name cited within a botanical paper is linked as well as vice versa. The PTP may be also used for names not cited within a paper and functions as a focused harvester without any charges or barriers to the readers.

**Table 1.** External web resources currently linked to taxon names cited within PhytoKeys papers, provided through the Pensoft Taxon Profile (PTP) (www.ptp.pensoft.eu) (see also Penev et al. 2010a).

*Source name*	*Web address*
**General sources**	
Global Biodiversity Information Facility	www.gbif.org
Encyclopedia of Life	www.eol.org
Catalogue of Life	www.catalogueoflife.org
ITIS	www.itis.gov
uBio	www.ubio.org
WoRMS – World register of Marine Species	www.marinespecies.org
BioLib	www.biolib.cz
Plazi	www.plazi.org
IUCN – International Union for Conservation of Nature	www.iucn.org
Wikipedia	www.wikipedia.org
Wikispecies	www.species.wikimedia.org
**Taxon-oriented sources**	
International Plant Name Index	www.ipni.org
Tropicos	www.tropicos.org
PLANTS Database	www.plants.usda.gov
The Gymnosperm Database	www.conifers.org
**Gene sequences**	
NCBI - National Center for Biodiversity Information	www.ncbi.nlm.nih.gov
Barcode of Life Data Systems	www.boldsystems.org
**Images**	
Morphbank	www.morphbank.net
Wikimedia	www.wikimedia.org
Yahoo	www.images.search.yahoo.com
**Literature references**	
Google Scholar	www.scholar.google.com
PubMed	www.ncbi.nlm.nih.gov/pubmed
BHL - Biodiversity Heritage Library	www.biodiversitylibrary.org

**Figure 2.**
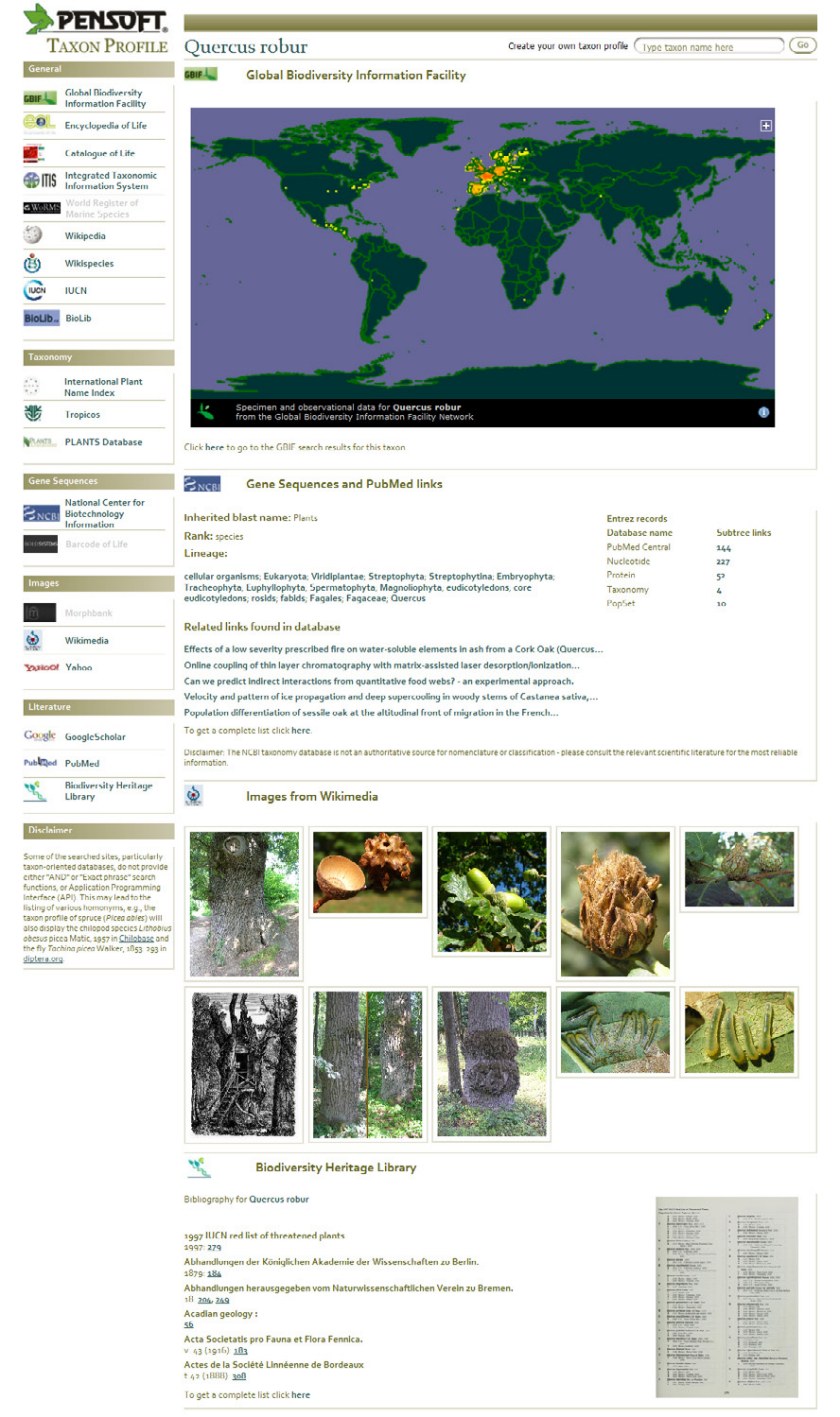
Dynamic webpage (taxon profile) of the the English oak (Quercus robur L. ) generated “on the fly” by the Pensoft Taxon Profile tool (PTP, http://ptp.pensoft.eu)

The mission of PhytoKeys is to constantly extend its cross-linking and linkout programs with the main aim being to harvest as many data as possible from biodiversity sources and thereby serving in this way our authors, reviewers, editors and readers.

## Editorial policies, focus and scope

The editorial policy of PhytoKeys will be based on the following principles:

- High quality of published papers, controlled by an eminent editorial board and rigorous peer-review process;
- Open access to all published content ensuring the widest possible barrier-free distribution of works at no charge for readers;
- Author copyright and distribution under the Creative Commons Attribution 3.0 license;
- Quick turn-around time, ranging between 3–6 weeks for review and 1–2 weeks for publication, after manuscript acceptance;
- Online submission and editorial management system, professional review and editorial assistance, typesetting, proofreading and publication;
- No limit in manuscript length; large revisionary works, checklists, catalogues, etc. will be published as special journal issues in the form of separate monographs with assigned ISBN numbers as well as the standard ISSN of the journal;
- Publication in four different formats: (1) high-resolution, full-colour print version (2) PDF identical to the printed version; (3) HTML to provide links to external resources and semantic enhancements to published texts for interactive reading, and (4) XML version for archiving in PubMedCentral thus providing a machine-readable copy of the content to facilitate future data mining;
- Continuous development and implementation of cutting-edge publishing technologies: XML-based editorial work flow and mark up process, data publication and various semantic Web 2.0 enhancements, such as linking of all taxon names to external sources (e.g., GBIF, EOL, IPNI, etc.), as well as linking references to the Biodiversity Heritage Library (BHL) and other bibliographic sources, gene sequences to Genbank and so on;
- Submission of all new taxa to the International Plant Name Index (IPNI) within a few days of publication;
- Automatically generated dynamic web pages for all taxonomic names mentioned within a publication, by linkout to a wide array of leading biodiversity sites, through the Pensoft Taxon Profile (PTP) tool;
- Publishing of species-by-occurrence datasets under separate DOI numbers and indexing of published datasets with GBIF, simultaneous with the publication process;
- Data section providing automated generation and mark up of manuscripts from the metadata catalogue of the Global Biodiversity Information Facilities (GBIF);
- Acceptance of manuscripts automatically generated in XML files from databases, e.g., Scratchpads and LifeDesks web platforms;
- Immediate Alert Service through Email and RSS feeds to inform interested colleagues and organisations about your publication;
- Immediate distribution and dissemination of your publication to scientific databases, indices and search engines (ISI Web of Knowledge, Google Scholar, CABI Abstracts, DOAJ, and others);
- Archiving of your publication, electronically and in print, in trusted (e-) archives and libraries, in the first case PubMedCentral.

One of the highest priority objectives of PhytoKeys will be quick coverage by ISI Web of Science and the assignment of an impact factor by the end of the second year of existence. The accumulated experience with ZooKeys gives us confidence that this goal is definitely achievable.

PhytoKeys will consider for publicationworks in taxonomy, systematics, biogeography, evolution, and phylogeny in the widest possible sense. Examples of such papers are new descriptions of taxa, if they are accompanied by proper diagnoses, keys and/or distinction from at least the related or similar species; taxonomic revisions of extant (or ‚‘recent”) and fossil plant groups; checklists and catalogues; phylogenetic and evolutionary analyses; plant DNA barcode analyses; papers in descriptive and/or historical biogeography; methodology papers; data mining and literature surveys; monographs, conspectus, and atlases; collections of papers, Festschrift volumes, and conference proceedings.

Ecological papers will be considered if they treat specific taxa or as part of special issues on a certain topic, region or taxon.

The following categories of papers will also be considered for publishing: original research articles; reviews as longer articles offering a comprehensive overview, historical analysis or/and future perspectives of a topic; monographs and collections of papers with no limit in size, published as “special issues”; data papers; short communications; letters and discussion papers; book reviews.

Authors and editors publishing large revisions or surveys, collection of papers, conference proceedings, Festschrift volumes, checklists, catalogues, etc. will benefit from being assigned ISBN numbers to their works, providing in this way additional dissemination and promotion through the book industry framework.

We are convinced that PhytoKeys will establish a new model of publishing and dissemination of information in botany taking advantage of the exciting possibilities in the application of the semantic Web. New technologies implemented in PhytoKeys will permit taxonomists, ecologists, conservationists and any reader anywhere to harvest within seconds the most essential information on a taxon, locality, or even a specimen, such as descriptions, images, maps, keys, gene sequences and references. A significant impediment to the acceleration of knowledge about the diversity of our planet is access to all the information accumulated during the long history of scientific discoveries; information published in PhytoKeys will be free and open access for anyone to read and use. We are committed to enhancing access to and speeding up the dissemination of taxonomic knowledge, and all efforts of PhytoKeys will be directed to advance knowledge about plant life on Earth.
